# The immunogenic radiation and new players in immunotherapy and targeted therapy for head and neck cancer

**DOI:** 10.3389/froh.2023.1180869

**Published:** 2023-07-11

**Authors:** Shay Sharon, Narmeen Daher-Ghanem, Deema Zaid, Michael J. Gough, Nataly Kravchenko-Balasha

**Affiliations:** ^1^ Department of Oral and Maxillofacial Surgery, Hadassah Medical Center, Faculty of Dental Medicine, The Hebrew University of Jerusalem, Jerusalem, Israel; ^2^ The Institute of Biomedical and Oral Research, The Hebrew University of Jerusalem, Jerusalem, Israel; ^3^ Department of Oral and Maxillofacial Surgery, Boston University and Boston Medical Center, Boston, MA, United States; ^4^ Earle A. Chiles Research Institute, Robert W. Franz Cancer Center, Providence Portland Medical Center, Portland, OR, United States

**Keywords:** head and neck cancer, immunotherapy, radiotherapy, SBRT, hypofractionated, targeted therapy, squamous cell carcinoma, HNSCC

## Abstract

Although treatment modalities for head and neck cancer have evolved considerably over the past decades, survival rates have plateaued. The treatment options remained limited to definitive surgery, surgery followed by fractionated radiotherapy with optional chemotherapy, and a definitive combination of fractionated radiotherapy and chemotherapy. Lately, immunotherapy has been introduced as the fourth modality of treatment, mainly administered as a single checkpoint inhibitor for recurrent or metastatic disease. While other regimens and combinations of immunotherapy and targeted therapy are being tested in clinical trials, adapting the appropriate regimens to patients and predicting their outcomes have yet to reach the clinical setting. Radiotherapy is mainly regarded as a means to target cancer cells while minimizing the unwanted peripheral effect. Radiotherapy regimens and fractionation are designed to serve this purpose, while the systemic effect of radiation on the immune response is rarely considered a factor while designing treatment. To bridge this gap, this review will highlight the effect of radiotherapy on the tumor microenvironment locally, and the immune response systemically. We will review the methodology to identify potential targets for therapy in the tumor microenvironment and the scientific basis for combining targeted therapy and radiotherapy. We will describe a current experience in preclinical models to test these combinations and propose how challenges in this realm may be faced. We will review new players in targeted therapy and their utilization to drive immunogenic response against head and neck cancer. We will outline the factors contributing to head and neck cancer heterogeneity and their effect on the response to radiotherapy. We will review *in-silico* methods to decipher intertumoral and intratumoral heterogeneity and how these algorithms can predict treatment outcomes. We propose that (a) the sequence of surgery, radiotherapy, chemotherapy, and targeted therapy should be designed not only to annul cancer directly, but to prime the immune response. (b) Fractionation of radiotherapy and the extent of the irradiated field should facilitate systemic immunity to develop. (c) New players in targeted therapy should be evaluated in translational studies toward clinical trials. (d) Head and neck cancer treatment should be personalized according to patients and tumor-specific factors.

## 1. Introduction

### 1.1. Head and neck cancer

Head and neck squamous cell carcinoma (HNSCC) accounted for approximately 878,000 newly diagnosed cases worldwide in 2020 (1), and approximately 68% of patients with oral cavity and pharyngeal cancer are expected to survive five years (2). The two conventional approaches to treating HNSCC are primary surgery followed by risk-adapted chemoradiotherapy or upfront definitive chemoradiotherapy. Chemotherapy for HNSCC is mainly based on high-dose cisplatin and fractionated radiation therapy (RT) delivered to a total of 66–70 Gy. For patients with advanced comorbidities or poor performance status, these approaches often lead to unacceptable treatment-associated morbidity and mortality. Recurrent or metastatic (R/M) HNSCC poses an even greater challenge as only one-third of patients respond to treatment, primarily chemoradiotherapy, and the median survival period is 6–8 months (3).

### 1.2. Radiotherapy

RT has evolved over the years, and more than ever, it targets cancer cells. Its design serves this purpose by utilizing the principles of radiation physics and fractioning into smaller doses. The conventional RT that most patients undergo is fractionated, during which small doses of radiation (around 2 Gy per fraction) are delivered daily. This method presumably allows for normal tissue to undergo repair better than tumor tissue (4), thus targeting the destructive radiation effect on cancer cells more than on their surrounding healthy counterparts.

An alternative fractionation method is based on delivering high-dose radiation in either a single dose or a limited number of doses. Defined as hypofractionation, or stereotactic body radiotherapy (SBRT), this method enables a high radiation dose to be focused on a specific location while maintaining a steep dose gradient beyond (5). For patients who are unable to withstand the prolonged fractionated RT regimen or surgery, primary SBRT has yielded impressive local control and overall survival (OS) rates while maintaining relatively low radiation-related adverse features (6–8).

### 1.3. The immunogenic radiation and SBRT

SBRT can be seen, like surgery, as an opportunity to focally treat a cancer site. For many years, the effect of RT on the immune system was generally perceived as immunosuppressive. It was backed by data showing lymphopenia, leukocyte cytotoxicity, and impaired leukocyte function in response to RT (9–12).

However, a growing amount of evidence supports an additional, synergistic effect of RT. The synergistic effect, under certain conditions, functions as an *in-situ* vaccine that primes the immune response both locally and systemically (13, 14), and drives the immune response to control distant disease (15). Preclinical models and clinical reports have linked the induction of the immune response by RT to the abscopal effect. This effect is evident when locally irradiating a primary tumor and consequently witnessing the regression of distant metastases outside the irradiated field (16, 17). This process clearly stems from a systemic response to radiation, pointing to the immune system as a potential key factor in this process.

**Figure 1:**
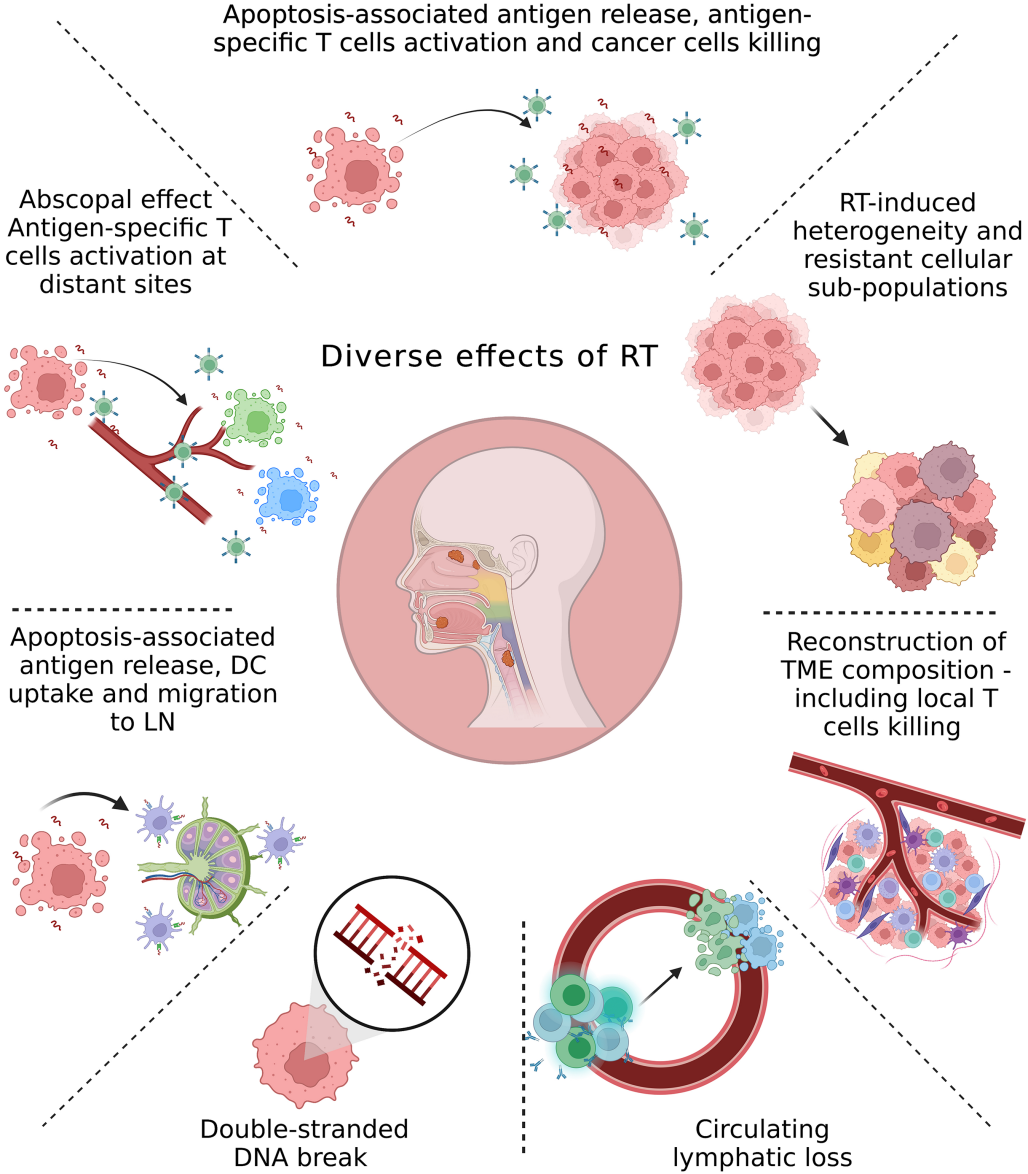
Overview of the main radiation-induced changes in the tumor environment and cancer cells. The local and systemic effects of irradiation can be linked to the radiation dose and fractionation. Although the initial effect of radiation therapy (RT) is cell death due to DNA damage, accumulating evidence from recent studies demonstrates multiple local and systemic molecular alterations induced by RT. These include antigen release and antigen-specific T-cells activation; molecular changes in the tumor microenvironment (TME) due to direct effects of RT or interactions between dying cells and the TME; RT-induced development of resistant subpopulations within the tumor; lymphocyte loss; abscopal effect; trafficking of dendritic cells (DC) from tumors to the tumor-draining lymph node (LN).

A different synergistic effect is demonstrated by the radiation-induced changes in the tumor environment and its surviving cancer cells to drive an immune-mediated local clearance of residual disease. Radiation-induced cell damage triggers the tumor to release antigens, which have the potential to generate new T-cells to attack the tumor with antigen specificities that were not formerly involved—termed epitope spreading (10, 18, 19) (Figure 1). However, antigen release alone is insufficient, as innate adjuvants are essential for effective immunity (20). In the sterile immunity of radiation-induced cell death, these innate adjuvants are endogenous adjuvants released by dying cancer cells (20, 21). Understanding the pattern of innate adjuvants released by dying cancer cells is critical, since some forms of cell death are differentially immunogenic (21–23). Similarly, the cell types that respond to these adjuvants and their differentiation dramatically impact the immune consequences of cancer cell death and adjuvant release (24–29). Thus, if the response to radiation is optimal, cancer cell death will release antigen and adjuvant to promote dendritic cells (DC) maturation to boost existing T-cell responses and generate new T-cell responses. It will also generate a pro-inflammatory environment in the tumor to help attract effector T-cells and guide myeloid differentiation into anti-tumor patterns. A suboptimal response will fail to mature DC (30, 31), and generate suppressive cytokine release from cells, such as M2-differentiated macrophages in the tumor environment (25).

The local and systemic effects can be linked to the radiation dose and fractionation, and no discussion of RT is complete without their appreciation. Basic radiobiology demonstrates that splitting doses into multiple fractions has non-linear effects on radiation-mediated cell death (32). This concept was demonstrated in the clinical setting after the introduction of SBRT into the field of intracranial tumors (33), and was followed by its application to extracranial sites using ablative doses of radiation (8–30 Gy per fraction) (4).

Since then, it was shown that SBRT could be both effective and well-tolerated in various types of cancer, including non-small-cell lung cancer (NSCLC) (34), with local control rates of above 90% at three years (35–37); and prostate cancer with progression-free survival (PFS) rates of 97% at five years (38). In contrast to lymphopenia that may be triggered by conventional RT, administering neoadjuvant SBRT concurrently with durvalumab (anti-PD1) in HNSCC led to increased white blood cell counts (39). We will review the immunological basis of dose and fractionation-dependent effects, and describe the related data from preclinical models and clinical trials in head and neck cancer.

### 1.4. Immunotherapy

Immunotherapy has been developing rapidly over the last decade. It has the potential to activate an immune response to target cancer cells by utilizing the function of the immune system to survey the body for abnormal cells and eliminate them continually. Cancer cells that were not eliminated can exist in equilibrium with the immune response until their eventual evasion from it, defined as immune escape (40). The balance may be tilted in favor of the immune response with targeted therapy.

Programmed cell death protein 1 (PD-1) is a receptor expressed on immune cells that drives the downregulation of the immune response (41) and is blocked by immune checkpoint inhibitors. Nivolumab and pembrolizumab, both PD-1 checkpoint inhibitors, were approved by the Food and Drug Administration (FDA) in 2016 as second-line treatment modalities for R/M HNSCC, following clinical trials demonstrating a favorable response in platinum-refractory R/M HNSCC (42, 43). Although the absolute number of months added to OS in these trials was limited, one must remember they were conducted in the R/M setting (42, 44–46).

In 2019, pembrolizumab was approved as first-line monotherapy for R/M HNSCC or in combination with chemotherapy. Due to the low response rates to monotherapy checkpoint inhibitors in R/M HNSCC, more clinical trials focus on combining immunotherapeutic agents, concurrent immunotherapy and chemotherapy, and concurrent immunotherapy and RT. However, the results of trials adding immunotherapy to the standard treatment for locally-advanced HNSCC have not been as promising as expected. The JAVELIN Head and Neck 100 trial tested the addition of a programmed cell death ligand 1 (PD-L1) inhibitor to chemoradiotherapy and was halted when the primary objective of prolonging PFS was not reached (47). The GORTEC 2015-01 PembroRad trial replaced high-dose cisplatin with pembrolizumab in combination with RT, and showed similar disease control rates (48). The outcomes of these trials underscore the complexity in assigning the most suitable treatment to a particular cancer patient and predicting its success (49).

## 2. The immunogenic radiation

### 2.1. Introduction to dose/fractionation-dependent effects

Technological developments in physics and computing have permitted highly conformal targeting of tumors while avoiding normal tissues. It allowed higher doses of radiation to be delivered in fewer fractions and optimization of RT-fractionation to permit tumor-selective death. Currently, most patients are treated with standard fractionation with individual doses at or below 2 Gy, and treatments are delivered over several weeks. In HNSCC, these doses add up to 66–70 Gy over 6–7 weeks and may be delivered concurrently with chemotherapy.

In preclinical models, however, a daily regimen of 2 Gy fractionation over multiple weeks is rarely tested (50). First, it stems from a practical limitation in the growth rate and timeline of murine models. Second, fractionated radiation negatively affects the proliferating T-cells, and studies are generally designed to avoid this effect. For example, in a model where high-dose RT (30 Gy) resulted in effective CD8^+^ T-cells anti-tumor response, adding fractionated RT (3 Gy ×10) decreased tumor control (51). These data demonstrate that extending the timeline of radiation treatment can kill T-cells which are critical for tumor control (52).

As for the radiation dose, this area of research has not reached a firm conclusion. In some cases, preclinical models show that higher radiation doses lead to optimal synergism with immune combinations. For example, 5–8 Gy per fraction has been successfully employed (53–55), which is consistent with the optimal dose which led to the release of immunological adjuvants from cancer cells following RT (56, 57).

Morisada et al. compared the effect of administering 2 and 8 Gy RT to mouse oral cancer cells *in-vitro* and *in-vivo*. 8 Gy induced greater tumor-cells susceptibility to T-cell cytotoxicity than 2 Gy, and dose-dependency was demonstrated in terms of antigen release, antigen-specific T-cells activation, and cytotoxic targeting of cells (58). The same group later showed enhanced anti-tumor immunity when administering hypofractionated RT (8 Gy ×2), compared to hyperfractionated RT (2 Gy ×10). When RT was combined with PD-1 blockade, better control of primary and distant tumors was achieved (59).

In contrast, other preclinical studies have shown that synergy with immunotherapy was achieved at low doses of RT (60–63).

Clinical studies are no different. While some use higher doses of radiation in combination with immunotherapy (64–66), others use conventional fractionation (67). Certain immunotherapies likely require specific radiation dose as well as an optimal timeline of delivery (68), each deserves preclinical modeling prior to its clinical translation (50).

### 2.2. Direct effects of radiation on immune cells in the field

T-cells are a dynamic population that recirculates in and out of tissues via the draining lymphatics and back into the peripheral blood (69). Hence, understanding the effect of RT on T-cells should encompass data from all sites: The peripheral blood, the tissues, and the draining lymphatics.

The clearest data relating to the impact of radiation on the immune profile of tumors is the direct radiation-mediated killing of T-cells in the treatment field [reviewed in (52)]. Systemic lymphocyte loss is another immune-related outcome observed in patients treated with conventionally fractionated radiation (Figure 1) (11, 12, 70–75), though alterations in dose and fractionation can limit this effect (76, 77).

Following total body radiation, T-cells in the tumor are relatively radio-resistant compared to circulating T-cells (78) but are still killed by focal radiation therapy (78, 79). This is critical since the tumor is enriched for tumor antigen-specific T-cells, and tumors with a more significant proportion of tumor-specific T-cells are associated with improved prognosis (80). If radiation of the tumor eliminated all tumor-specific T-cells, then it would likely be a poor partner for T-cell-targeted immunotherapies (52). However, since T-cells recirculate in and out of tissues via lymphatics and back into the peripheral blood (69), a transient local loss of T-cells following treatment can be recovered by infiltration from the circulation, and local proliferation (81). Notably, irradiation of T-cells in the tumor-draining lymph node can impair reconstitution and impair tumor control by radiation and some immunotherapy combinations (82). These data suggest that reconstitution from some non-tumor sources is critical to the success of some radiation and immunotherapy combinations.

*In-vitro* studies exposing circulating blood cells to low doses of RT *ex-vivo* have demonstrated that a significant increase in T-cell death was detectable at 0.125 Gy, and approximately half of the T-cells underwent apoptosis at 2 Gy (83). By contrast, myeloid populations were relatively radio-resistant at these doses (83, 84). Among myeloid populations, DC and Langerhans cells have been shown to be more radio-resistant than T-cell populations (83, 85–87). When monocytes are differentiated into DC *ex-vivo*, the cells become less radiosensitive (83), in part due to the terminally differentiated and non-proliferative status of DC (85, 86, 88). However, DC can be directly impacted by radiation. Human DC given high-dose radiation (30 Gy) demonstrated a suppressed co-stimulatory phenotype and MHC Class II expression (89). Irradiation of murine bone marrow-derived DC has been shown to affect antigen-presentation pathways and their ability to generate T-cell responses following injection into mice (85). Irradiation (30 Gy) of human monocyte-derived DC resulted in inhibited IL-12 secretion and reduced ability to stimulate T-cells, but phagocytosis and migration were not impaired (88). Similarly, radiation doses above 6 Gy resulted in decreased IL-23 secretion and decreased *ex-vivo* Th17 priming ability (90). These data suggest that while DC are radio-resistant, they are susceptible to radiation-mediated direct effects.

It is important to note that these studies of DC use higher doses than are typically used for *in-vitro* studies, and in *ex-vivo* studies, the positive impact of radiation-induced adjuvant release is not well modeled. Using a fluorescence model to label infiltrating cells in murine tumors, we observed the trafficking of dendritic cells from tumors to the tumor-draining lymph node (30). Treatment of the tumors with 12 Gy resulted in migration and maturation of DC in radio-immunogenic tumor models but not in poorly radio-immunogenic models (30). The difference between these models is that the poorly radio-immunogenic model had minimal T-cell involvement in tumor control following radiation (24, 31), suggesting that DC migration is a potential reason for the discrepancy. However, since each model was treated with the same 12 Gy radiation dose, it demonstrates that DC can be fully functional when exposed to high single doses of RT *in-vivo* and can be a critical cell type to propagate immune responses following RT.

### 2.3. The immune effect of treating lymph nodes of the neck

Elective neck irradiation is frequently employed to irradicate microscopic disease in the draining lymph nodes of the neck. However, as lymph nodes are the site in which tumor-specific T-cells reside, antigen-presenting cells are primed, and central memory is established (91–95), irradiation of the draining lymph nodes may hinder the immune response and the effect of immunotherapy.

Using an *in-vivo* model of HNSCC, Darragh et al. administered 8 Gy ×3 to the primary oral tumor, with or without elective nodal irradiation. The resulting data showed that sparing the neck led to better local control, better distant control, induction of epitope spreading, increased activation of CD8^+^ T-cells, and no lung metastases. However, while sparing the neck led to better local and distant control, regional recurrence was observed only in this group. Elective neck dissection performed five days after tumor cells implantation demonstrated similar results to elective nodal irradiation (91). These data suggest that while maintaining the integrity of the draining lymph nodes may induce immune response propagating to local and distant control, their persistence may eventually lead to regional recurrence. Notably, removing the sentinel lymph nodes was sufficient to prevent regional recurrence (91). Thus, the timing of surgery relative to immunotherapy and RT is critical. While upfront surgery followed by an immunotherapy-RT combination led to worse local control and immune response, a neoadjuvant immunotherapy-RT combination followed by surgery resulted in better local control and systemic immunity. This benefit was maintained even if the neck lymph nodes were removed, either entirely or by removing sentinel lymph nodes alone, as long as they were removed after administering immunotherapy and SBRT to the primary tumor (91).

Similar results were observed when checkpoint inhibitors were preceded by neck dissection or high-dose neck RT in murine orthotopic tongue tumors, both significantly reducing OS (96). The tumor-draining lymph nodes were found to be the critical component for immune checkpoint inhibitors response after ipsilateral, and not contralateral, neck dissection led to a compromised response to immune checkpoint inhibitors in a lateralized orthotopic model (96). A significant increase in CD45-negative cells and a decrease in the amount of CD8^+^ and CD4^+^ T-cells within the tumor was observed in the neck dissection group (96). Complete response of murine orthotopic tongue tumors was achieved following anti-CTLA-4 (cytotoxic T-lymphocyte–associated antigen 4) or anti-PD-1, and this effect was unchanged following a subsequent late (+6 days) neck dissection. However, an early neck dissection (+1 day) hindered the complete response (96). These data suggest that administering immunotherapy should commence in the neoadjuvant setting, and that neck irradiation or dissection should be timed late enough to allow systemic immunity to develop.

### 2.4. Cancer cell death as a source of antigen and adjuvant

Many other changes that occur in the tumor immune environment following radiation are secondary to the effects of radiation on cancer cells (Figure 1). The primary focus of RT is cancer cell death, which necessitates phagocytic clearance, a defining feature of the immune response in the vicinity of dying cells (22, 97, 98). The interaction of dying cancer cells with phagocytic myeloid cells in the tumor environment can drive their differentiation into suppressive states that limit the immune control of tumors following radiation (99, 100). However, DC uptake and cross-presentation of tumor-associated antigen is critical for subsequent T-cell responses in the tumor-draining lymph nodes (101).

This cross-presentation of cell-associated antigen to T-cells provides *Signal 1* to T-cells via cognate interaction with the T-cell receptor. *Signal 2* is an essential second step in T-cell activation provided by the antigen-presenting cell in response to adjuvant signals in their environment. *Signal 2* is delivered by costimulatory molecules such as CD80 and CD86 that are induced on antigen-presenting cells following their exposure to innate adjuvants, as well as following antigen presentation to CD4^+^ T-cells (29, 102–105). In the case of infectious disease, these immunological adjuvants are bacterial or viral components directly recognized by Toll-Like Receptor or similar pathways in the antigen-presenting cell. T-cells receive *signal 2* through CD28, which synergizes with TCR ligation to activate critical activation pathways in the T-cell (106–110). *Signal 1* without *signal 2* can tolerize T-cells or result in their deletion (111, 112), so without immunological adjuvant release, cancer cell death is potentially able to delete tumor antigen-specific T-cells.

Importantly, as we will discuss later, a range of immunological adjuvants being released following radiation-mediated cancer cell death *in-vitro* and *in-vivo* have been described (20, 21, 113, 114). Together, cancer cell death following RT has the potential to provide *signal 1* to T-cells following antigen release from dying cells via antigen delivered to cross-presenting DC, and *signal 2* in the form of endogenous adjuvants. This can cause DC maturation, migration to the draining lymph nodes, and upregulation of CD80 and CD86. In this way, DC are the immunological mechanism that links cancer cell death to *signal 1* and *signal 2* in T-cells. Notably, the degree of DC maturation varies between preclinical tumor models exposed to identical RT (31), which in turn affects DC migration to the tumor-draining lymph node and, therefore, T-cell involvement in the control of residual disease following radiation (24, 30). Factors contributing to this heterogeneity in radiation response and methods to analyze heterogeneity will be discussed later.

### 2.5. The abscopal effect

Other than the direct effects of radiation on immune cell types discussed above, a large portion of the early work in the immune aspect of RT resulted from studies exploring the mechanisms of post-radiation fibrosis, and the elusive abscopal effect. The abscopal effect is evident when locally irradiating a primary tumor and consequently witnessing the regression of distant metastases outside the irradiated field (16, 17). In fibrosis, immune cells, cytokines, and growth factors underlie the transition from cell death following radiation to abnormal repair in field (115, 116). In studying the abscopal effects, it was necessary to find mechanisms that could support radiation-mediated cell killing in the treated tumor and act at a distance, and a range of angiogenic and cytokine mediators were initially proposed (117–121). Thanks to pioneering studies, it is now appreciated that abscopal effects can be mediated by T-cells (14), which through recirculation, can move between distant sites, including distant tumors (69).

### 2.6. The oligometastatic status

In 1995, Hellman & Weichselbaum defined the oligometastatic status as an intermediate condition on a spectrum extending from localized disease to a rapidly advancing systemic disease. On the one hand, the limited spread of metastases to the lymph nodes may be perceived as an aggressive disease since the involved nodes harbor cancer cells and are, thereby, a source for seeding cancer (122). On the other hand, albeit an advanced disease, the oligometastatic status has not yet progressed into a systemic state, so it can be potentially cured.

To allow for clinical decision-making, the European Society for Radiotherapy and Oncology (ESTRO) and the European Organisation for Research and Treatment of Cancer (EORTC) proposed a consensus for classifying and characterizing the oligometastatic disease. It was based on the first cohort of the ESTRO and EORTC OligoCare registry project, focusing on differentiating between oligometastatic states and subclassifying the oligometastatic disease into oligorecurrence, oligoprogression, and oligopersistence (123). Although this system requires further evaluation, it underlines the importance of perceiving the oligometastatic disease as a heterogeneous state that needs to be addressed as such.

Currently, HNSCC patients with distant metastasis are classified as M1 and treated systemically. However, surgical removal of a metastasis (metastasectomy) or targeting it with stereotactic ablative radiotherapy (SABR) is employed in certain types of cancer. Metastasectomy was beneficial for lung metastases, prolonging life, and potentially curative in a selected group of patients (124).

By introducing the definition of oligometastatic disease into the diagnostic process, metastases originating from the lung, adrenal, liver, and spine may be targeted by SABR/SBRT (125). In a study that concentrated on delivering SABR to lung oligometastases originating from HNSCC, Bates et al. showed that although the 2-year disease-free survival was only 14%, the 2-year OS was more encouraging, reaching 43% (126). These data suggest that targeting oligometastases with SBRT should be considered to improve OS in HNSCC. Whether adding targeted therapy to prime the immune response to irradiation of oligometastases will improve OS and disease-free survival remains to be seen in future trials.

### 2.7. Combining radiation and immune checkpoint inhibitors in preclinical models

The combination of RT and immunotherapy is gaining interest as an avenue for cancer treatment, as recently reviewed (49, 127–129). Although limited initially, data on the combination of RT and immunotherapy in preclinical models is now accumulating. Using preclinical models allows for the testing of combinations regimens and mechanistic interventions that help understand how treatments impact tumors, both not practically feasible in large-scale clinical trials (50). Selecting the optimum immunotherapy to combine with radiation may depend on the immune status of the patient's tumor. For example, immunotherapies that target exhausted T-cells, such as checkpoint inhibitors, will likely be most successful where the patient has an extensive immune infiltrate limited by expression of these checkpoint molecules. By contrast, where a patient lacks extensive pre-existing immunity, the optimum immunotherapy may be better targeted to initiate anti-tumor immune responses in the tumor-draining lymph nodes, focusing on DC-related innate adjuvants (29–31), or costimulatory molecules such as ICOS and OX40 that are induced following antigen exposure (130, 131). A range of immune interventions in combination with radiation are discussed below.

Given the T-cell mechanism of action, it becomes logical to deliver therapies that act on T-cells to improve local and distant tumor control following radiation. Currently, almost all candidate T-cell targeted immunotherapies have shown synergy with radiation in some preclinical settings. The dominant players have been anti-CTLA-4 and anti-PD1.

Inhibition of CTLA-4 in combination with irradiating mouse primary mammary tumor led to an anti-tumoral immune response which inhibited the formation of lung metastases (132). To test dose fractionation that induces an abscopal effect, breast carcinoma tumors were implanted *in-vivo* in two separate sites and treated with different combinations of systemic CTLA-4 blockade and RT targeted to a single tumor site in a range of doses and fractions. By following the irradiated and unirradiated tumors, the experiments allowed an assessment of local control and abscopal effects, respectively. Both the fractionated and single-dose regimens caused a delay in the growth of the irradiated tumor, and the addition of anti-CTLA-4 further enhanced the effect. However, the non-irradiated tumor exhibited growth delay only in mice treated with the combination of fractionated radiation and CTLA-4 blockade (55). These data suggest that fractionated RT is a better partner for CTLA-4 blockade than single-dose RT to generate an abscopal effect. Combining RT and CTLA-4 blockade in a similar model also demonstrated a significant survival benefit (132).

Using the murine pancreatic ductal adenocarcinoma model, it was shown that the addition of anti-PD-L1 to high-dose RT improved tumor response and further prevented the development of liver metastases. This effect was evident following hypofractionated high doses of radiation but not after using low-dose radiation (133). Combining a single dose (10 Gy) of SBRT and PD-1 blockade gave rise to a significant long-term survival advantage in the orthotropic mouse glioma model; mice treated with PD-1 blockade or SBRT as monotherapy did not exhibit a significant advantage over the untreated group (134). A combination of PD-1 blockade and SBRT induced near-complete regression of irradiated mouse melanoma and partially reduced the size of the non-irradiated tumor. This effect was less prominent in the sole blockade of PD-1 or the RT-only groups (135).

The importance of timing and fractionation regimen was studied using the murine colon carcinoma and breast cancer models; blocking PD-1 or PD-L1 enhanced the efficacy of RT, while fractionated RT upregulated PD-L1 expression. Importantly, the highest efficacy was noted in the concurrent RT/PD-L1 blockade arm but diminished after delaying PD-L1 blockade for five days and became virtually non-beneficial following PD-L1 blockade initiated seven days after completion of RT (60). A single 10 Gy dose to an orthotopic model of HNSCC led to the upregulation of PD-L1 on tumor cells and increased T-cell infiltration, thereby improving local control and OS (136). Integration of single-dose RT with immunotherapy has also upregulated the expression of murine PD-L1 in the tumor microenvironment (TME), and blockade of PD-L1/PD-1 in combination with radiation showed a cumulative positive effect (137).

Notably, the combination of CTLA-4 blockade and PD-1/PD-L1 blockade provides distinct synergy with radiation such that the combination is more effective than any alone (138). Other T-cell targets that have shown preclinical efficacy include a range of TNFRSF members, including LIGHT (54), OX40 (131), 41BB (139), GITR (140), and ICOS (130), as well as alternative targets showing efficacy in combination such as Tim3, TIGIT, and Lag3 (141, 142).

These data establish the basis for the RT-immune checkpoint inhibitors combination and the importance of choosing a radiation dose, fractionation, timing, and sequencing with immune checkpoint inhibitors.

## 3. Radiotherapy and immunotherapy in clinical trials

### 3.1. From conventional radiotherapy to SBRT

Previously, SBRT in HNSCC was mainly used in patients unable to tolerate the prolonged course of RT due to comorbidities, advanced age, poor social and financial support, or inability to travel daily (143, 144). Thus, studies mainly concentrated on utilizing SBRT for second primary tumors, reirradiation (145), or in recurrent or metastatic settings (146–150), and rarely as an upfront modality for newly diagnosed patients (151).

SBRT was also applied to boost conventional RT, mainly in nasopharyngeal and oropharyngeal cancers (152–154). Lee et al. described high 1-year and 2-year locoregional recurrence-free rates of 91.4% and 86.3%, respectively (*n* = 26). However, there was also a high frequency of acute complications (27%) and severe late complications (34.6%), which was more frequent among individuals who received concurrent chemo-RT two weeks prior (153). These data demonstrate the importance of fractionation dose and timing to the development of treatment-related toxicities.

Earlier studies reported the use of SBRT in patients unfit to undergo standard-of-care. Amini et al. described three patients aged 72–88 treated with 5.0–7.2 Gy ×5 and witnessed either a clinical or radiographic complete response, with no grade 3 toxicities or greater, at 4–8 months of follow-up (155). Khan et al. described 24 sites in 21 patients aged 25–103 (median 87), most of them diagnosed with SCC, who were treated with 4–6 fractions of 7–8 Gy and exhibited 25% complete response and 67% partial response at eight months of follow up (6). It was uncertain, however, whether SBRT could be used in place of fractionated RT and lead to comparable outcomes.

To test the potential of SBRT for reirradiation of recurrent or second primary head and neck cancer (squamous cell carcinoma in most patients), Vargo et al. retrospectively compared SBRT (*n* = 197) to intensity-modulated radiation therapy IMRT (*n* = 217). The two groups had different characteristics at baseline, with patients in the SBRT group being older and more heavily treated, more likely to be treated for recurrence than a second primary, and having more lifetime doses of RT. Although the unadjusted 2-year overall survival (OS) and median survival were higher among the IMRT group, after controlling for baseline differences, there was no difference between the groups in OS (HR 0.877; 95% CI: 0.702–1.097; *p* = 0.251) or cumulative incidence of locoregional failure (HR 1.154; 95% CI: 0.886–1.505; *p* = 0.289). A subset analysis, however, revealed that the OS of the two groups was similar as long as the tumor volume was small and the SBRT dose was ≥35 Gy. Otherwise, IMRT led to better OS. Patients in the IMRT group had a higher rate of acute grade ≥4 toxicity than in the SBRT group (5.1% vs. 0.5%, *p* < 0.01) (156). Thus, SBRT may be considered for patients with small tumors and should be administered to a total dose of no less than 35 Gy. These benefits are highlighted when compared to previous clinical trials investigating salvage reirradiation. Under the same settings, salvage reirradiation with conventional RT resulted in a median OS of 8.5 months in RTOG 9610 (157) and 12.1 months in RTOG 9911 (158). These trials showed grade ≥3 acute toxicity in 63%–78% and late toxicity in 22%–37% (157, 158). In clinically negative neck (N0) cases, locally recurrent and previously irradiated head and neck cancer patients pose a significant therapeutic challenge (159). Due to the limited data available on the potential benefits of surgical treatment for the N0 neck, it is important to investigate whether neoadjuvant targeted therapy can provide advantages in managing this challenging group of patients.

Considering its lower toxicity rate and comparable outcome to IMRT under certain circumstances, studies focused on identifying the factors contributing to better outcomes following SBRT. Comparing previously-irradiated with never-irradiated patients was among the main factors examined. To evaluate the effect of previous irradiation on treatment outcomes following SBRT, a retrospective review of unresectable head and neck cancer in medically unfit patients was carried out (*n* = 114, squamous cell carcinoma *n* = 81, skin primary *n* = 41, non-skin primary *n* = 59). Patients received a total dose of 35–50 Gy SBRT divided into 4–6 fractions and stratified according to their baseline disease status. There was a statistically significant difference in median progression-free survival (PFS) between the groups: 23.7 months (untreated primaries), 14.8 months (recurrent unirradiated primaries), 10.5 months (metastatic non-head and neck cancer primaries), and 7.8 months (recurrent irradiated head and neck cancer primaries) (*p* = 0.04). Although the local control in the recurrent irradiated primaries at 12 months (78.9%) did not significantly differ from other groups, both the PFS (7.8 months) and the locoregional recurrence rate (38.4%) were the worst among the recurrent irradiated primaries compared to the other groups. Indeed, multivariate analysis showed that the only significant variable was previously irradiated lesions, which were more likely to have shorter PFS than previously unirradiated lesions (HR 4.09, *p* = 0.03) (160).

These data align with a previous publication by Kodani et al. (*n* = 34), who observed a superior OS rate in SBRT-treated patients who have not undergone a prior RT within the previous two years or in cases of reduced target volume. In the same group of patients, 17.6% experienced severe late complications, all having a history of prior RT (161).

In a more recent publication focusing on previously unirradiated head and neck cancer patients unfit to standard-of-care (*n* = 66, SCC *n* = 44), 7–8 Gy ×5 SBRT was delivered biweekly. Thirty-four patients also received adjuvant therapy. Median time to local failure was 28.3 months, and 1-year local control and OS rates were 73% and 64%, respectively. The toxicity rate was low, with 3% grade 3 and no grade 4 or above toxicities (8). Compared to smaller studies, this larger study demonstrated similar OS and local control and reiterated the role of SBRT in patients unfit to undergo surgery and standard-of-care chemoradiation.

### 3.2. Combination therapies of SBRT, other than immunotherapy

Given the benefit of combining RT with chemotherapy and the systemic therapy administered in the recurrent or metastatic setting, the next step was to study the effect of combining SBRT with other modalities. Recurrent head and neck cancer patients (total *n* = 137, squamous cell carcinoma *n* = 98) were treated in a single institution with 4–5 fractions of SBRT to a total mean dose of 45 Gy (range 36–47.5 Gy) on an every-other-day schedule (median follow up 19.3 months) (145). This regimen resulted in OS of 78% (1-year) and 62% (2-year), and local, regional, and distant control of 78%, 66%, and 83%, respectively. Importantly, among patients who had disease progression, OS was significantly improved in those who received salvage therapy (surgery, RT, or systemic), compared to patients who did not (median OS 44.3 months vs. 15.3 months, *p* = 0.03). Concurrent systemic therapy was associated with increased regional control (73% vs. 53%, 1-year, *p* = 0.004) (145). Taken together, these data demonstrate the advantage of SBRT over conventional RT in treating recurrent head and neck cancer, and the benefit of combining SBRT with concurrent systemic therapy in these patients.

The combination of SBRT with cetuximab was the focus of many studies, including clinical trials. In a phase II clinical trial conducted in inoperable locoregional confined recurrent HNSCC (*n* = 50, median follow-up 18 months), the combination of SBRT (8–8.8 Gy ×5) and cetuximab was tested. The median OS was ten months (95% CI: 7–16), the median PFS was seven months (95% CI: 5–12), and the 1-year OS was 40% (95% CI: 26%–54%). Although the primary efficacy in this study was not met, the PFS was similar to conventional fractionated RT combination with cetuximab but with lower toxicity rates (149). Similar results were obtained in a multi-institutional phase II clinical trial testing the combination of SBRT (6 Gy ×6) and cetuximab in recurrent HNSCC (*n* = 60, median follow-up 11.4 months). The median PFS was 7.1 months (95% CI: 5.5–8.9), and the 1-year OS was 47.5% (95% CI: 30.8–62.4) (150). Although SBRT showed no benefit in OS in these populations over fractionated RT, it required a shorter overall treatment time and led to lower toxicity rates.

### 3.3. SBRT to metastases

Conventional RT in metastatic HNSCC was previously seen as a palliative measure, with hypofractionated RT also utilized for symptom relief (162). However, SBRT may generate impressive local control of metastatic disease. In a multi-institutional retrospective registry analysis of SBRT for the management of HNSCC, lung, non-regional lymph nodes, and spine metastases, 1-year and 2-year OS rates were 66.4% (95% CI: 53.4%–76.4%) and 43.1% (95% CI: 30.3%–55.2%), respectively, and local control rate was 93.3% (95% CI: 75.4%–99.3%) at 1-year and 2-year, and 76.4% (95% CI: 44.7%–91.4%) at three years (163). Due to the retrospective nature of this registry analysis, much data was missing, including the extent of disease, HPV status, the intent of RT (palliative vs. local control), and concurrent systemic treatment. However, it showed that using SBRT may induce local control of metastasis. Although there was a high variability of RT regimens and fractionation doses (6–22 Gy per fraction over 1–5 doses), and no correlation was identified between local control and either prescription dose or fractionation schedule, local control was notably higher in smaller metastatic lesions and lack of spinal osseous metastatic disease (163).

### 3.4. Summary—SBRT without immunotherapy

This body of data supports using SBRT as an alternative to fractionated RT under certain circumstances. First, it remains advantageous for medically unfit patients who cannot withstand standard-of-care treatment. Second, it should be considered in cases where the gross tumor volume (GTV) is small. GTV < 15 cm^3^ was associated with better OS in (161); recurrent GTV < 25 cm^3^ was associated with improved 1-year locoregional PFS (53% vs. 22%, *p* = 0.029) and 1-year OS (70% vs. 22%, *p* < 0.001) in (149); and ≤50 ml was associated with better median OS and PFS (21.9 and 19.1 months for ≤50 ml, 12.6 and 12.1 months for 50–100 ml, 8.6 and 8.6 months for >100 ml, respectively) in (164). Thirdly, in re-irradiated patients, an interval greater than one year since the previous irradiation correlated with better survival (157) and favorable treatment response (164), and greater than two years in (161, 165). Fourth, analysis of a national cancer database on SBRT for HNSCC showed that combining SBRT with surgery or chemotherapy yielded better OS than administering SBRT as a monotherapy (166). Lastly, better OS was associated with a fractionation regimen of 7 Gy ×5 or greater (166).

Ongoing SBRT clinical trials are summarized in Table 1.

**Table 1:** Selected SBRT clinical trials in head and neck cancer.

NCT ID	Title	Phase	Patients enrolled	HNSCC population	Number of SBRT fractions	Total dose
NCT02158234	SBRT and Concurrent Cisplatin for Re-Irradiation of Unresectable, Recurrent HNSCC	1	20	Re-irradiation of unresectable and recurrent	5	30 Gy
NCT05674396	3–5 fraction SBRT for Palliation of HNSCC: the FAST Phase II Randomized Trial	2	108	Ineligible for curative-intent treatment	3 to 5	
NCT03070366	SBRT Combined With Chemotherapy or Not for Treatment of Oligometastases in HNSCC (SBRT + Cx vs SBRT alone)	2	78	Oligometastatic	3 or 5	30/33/45 Gy or 35/50 Gy
NCT04435938	A Study of SBRT for HNSCC	2	38	Surgery and standrard RT not recommended/performed	5	45 Gy
NCT02057107	SBRT With Cetuximab ± Docetaxel Followed by Adjuvant Cetuximab +/- Docetaxel in Recurrent, Previously-Irradiated HNSCC	2	92	Recurrent, previously-irradiated	5	44–50 Gy

HNSCC, head and neck squamous cell carcinoma; SBRT, stereotactic body radiation therapy.

### 3.5. Combination of conventional radiation therapy and immunotherapy

The first phase III clinical trial to test the addition of a checkpoint inhibitor to chemoradiotherapy was the JAVELIN Head and Neck 100 trial. It was a placebo-controlled double-blind phase III study (*n* = 697) of locally advanced HNSCC patients treated with definitive fractionated RT and chemotherapy combination, and randomized to receive the anti-PD-L1 inhibitor avelumab (*n* = 350) or a placebo (*n* = 347). The trial was discontinued after the primary objective of prolonging PFS with avelumab was not reached (median PFS: 95% CI: 16.9 months-not estimable for avelumab, 23.0 months-not estimable for placebo), and an HR favoring the placebo group (HR 1.21; 95% CI: 0.93–1.75, *p* = 0.92) (47).

Given it was the first phase III trial to test the addition of immunotherapy to chemoradiotherapy in the upfront setting for locally advanced HNSCC, comparing it to other studies is challenging. However, based on other trials showing more favorable results following the addition of immunotherapy to chemotherapy without RT (46, 167), it is worth considering a possible negative effect of concurrent fractionated RT on the immune response. Moreover, the irradiated field in this trial included the neck draining lymph nodes, which might have impeded the priming of T-cells and hindered the effect of immune checkpoint inhibitors (91, 82, 96, 168).

Another trial focused on RT-immunotherapy combination is the phase II multicenter GORTEC 2015-01 PembroRad trial, which enrolled patients with locally-advanced HNSCC. This trial, however, recruited patients unfit to high-dose cisplatin, so it was not administered. Patients were randomized into pembrolizumab-RT (*n* = 67) vs. standard-of-care cetuximab-RT (*n* = 66) combinations, with a median follow-up of 25 months. Most patients had the oropharynx as the primary site (62% and 59%, cetuximab-RT and pembrolizumab-RT, respectively), and the minority had the oral cavity as the primary site (8% and 6%). Fractionated RT was administered in 33 daily fractions to a total dose of 69.96 Gy or 52.8 Gy. Three concurrent doses of pembrolizumab were administered at 3-week intervals. Both regimens achieved similar 15-month locoregional control (60% vs. 59%, pembrolizumab-RT vs. cetuximab-RT, respectively), and there was no significant difference in PFS (HR 0.85, 95% CI: 0.55–1.32; *p* = 0.47) or OS (HR 0.83. 95% CI: 0.49–1.40; *p* = 0.49). Although there was no statistically significant difference in PFS or OS, both trended in favor of the pembrolizumab-RT combination. Notably, the pembrolizumab-RT combination led to a statistically significant lower toxicity rate, with 74% vs. 92% of patients with adverse events ≥ grade 3 (*p* = 0.006) (48). These data suggest that pembrolizumab-RT may be a less toxic alternative to a high-dose cisplatin-RT combination, while still achieving similar OS and PFS. However, the neck was included in the irradiated field, and considering the data pointing to the possible role of an intact neck when commencing immunotherapy, a study designed to deliver immunotherapy-RT combination while limiting neck RT is the natural next step. The phase II REWRITe clinical trial in HNSCC (NCT03726775) will evaluate the combination of durvalumab and RT, restricted to the primary tumor and the adjacent neck levels.

These trials focused on administering immunotherapy and fractionated RT at the definitive setting. Given the potential of SBRT to induce an immune response, clinical trials are testing the combination of SBRT and immunotherapy in the metastatic setting.

### 3.6. SBRT-immunotherapy combination in the metastatic setting

An ample amount of data in solid tumors other than HNSCC emerges from combining SBRT and CTLA-4 blockade. Prescribing ipilimumab (anti-CTLA-4) and a single fraction 8 Gy RT in metastatic castration-resistant prostate cancer had not produced significant superiority over single-arm treatment (169). However, more encouraging results came from a study of solid metastatic tumors refractory to standard therapy. Five cohorts of patients were administered concurrent or sequential ipilimumab with 12.5 Gy ×4 or 6 Gy ×10 RT. Results indicated a possible correlation between an early increase in peripheral CD8^+^ T-cells, expression of 4–1BB and PD-1 on CD8^+^ T-cells, and a possible clinical benefit (170).

Concurrent delivery of ipilimumab and radiosurgery (median 21 Gy in 2 fractions) to melanoma brain metastases produced favorable regional control and amount of time to brain metastases progression, as opposed to the RT alone group (171, 172). However, in a different study, no superiority of immunotherapy in combination with RT was apparent over RT alone (173).

On the one hand, these data demonstrate a limited benefit at best; on the other hand, there is still no considerable amount of published data regarding this combination, and the already published data represents primarily studies conducted in advanced cancer populations.

In a single-center phase II trial (*n* = 62, median follow-up 20.2 months), a possible synergy between SBRT (9 Gy ×3) and anti-PD-1 immunotherapy was assessed in the metastatic HNSCC setting. Patients were randomized into nivolumab alone (*n* = 30) or nivolumab-SBRT combination (*n* = 32). At 12 months, there was no statistically significant difference in PFS (32.2% nivolumab; 95% CI, 19%–54.9%; 16.8% nivolumab-SBRT; 95% CI: 7.2%–39.3%), nor in median OS (14.2 nivolumab, 13.9 nivolumab-SBRT), overall response rate (34.5% nivolumab, 29.0% nivolumab-SBRT) or grade 3–5 toxicities rates (13.3% nivolumab, 9.7% nivolumab-SBRT; *p* = 0.7) (174). This trial showed no benefit to SBRT when added to nivolumab in the metastatic setting. To test whether concurrent targeting of the PD-1 and CTLA-4 pathways in addition to SBRT will lead to a benefit, the phase I/II clinical trial (NCT03283605) will administer both CTLA-4 (tremelimumab) and PD-1 (durvalumab) inhibitors concurrently with SBRT to metastatic head and neck carcinoma (*n* = 35, 2–10 extracranial metastases) (175). Another possibility is that this combination of SBRT and immunotherapy is insufficient to counteract the immune escape inherent to the metastatic state.

### 3.7. Neoadjuvant immunotherapy

The combination trials described thus far focused on administering immunotherapy and SBRT at the definitive setting, either as a concurrent treatment to standard-of-care or replacing chemotherapy. However, a growing body of data points to a potential benefit of administering immunotherapy in the neoadjuvant setting.

In a phase II randomized clinical trial (*n* = 29), neoadjuvant immunotherapy prior to surgical resection of oral cavity SCC resulted in a major to complete pathologic response in 8% (*n* = 1) of patients treated with neoadjuvant nivolumab, and in 20% (*n* = 3) of patients treated with neoadjuvant nivolumab + ipilimumab. Pathologic response greater than 50% was observed in 15% of patients receiving neoadjuvant nivolumab and 33% receiving nivolumab + ipilimumab. Pretreatment CD4^+^ T-cells were associated with pathologic response in the nivoloumab + ipilimumab combination but not in nivolumab alone (176). In a similar HNSCC non-randomized phase Ib/IIa clinical trial (*n* = 32), major pathologic response (MPR) was observed in 17% (*n* = 1) following nivolumab monotherapy and 35% (*n* = 8) following nivolumab + ipilimumab combination therapy. There was a trend of higher baseline intratumoral CD8^+^ T-cells density among major-pathological responders, albeit not statistically significant (*p* = 0.31). Interestingly, in both major-pathological responders and non-responders, there was an increase in intratumoral CD8^+^ T-cells density after neoadjuvant immunotherapy (177). Neoadjuvant pembrolizumab (*n* = 36, phase II trial, HPV-unrelated HNSCC), however, did not result in a complete response, and a major pathologic response was only evident in two patients (178).

Some studies showed a positive correlation between high-expressing PD-L1 populations and clinical outcomes following treatment with PD-1 inhibitors (42, 46, 47, 167, 179). However, while T-cells expressing PD-1 may decrease in responders post-treatment, baseline cell-specific expression of PD-L1 or combined positive score (CPS) do not necessarily differ between responders and non-responders to SBRT and anti-PD1 combination (39), or fractionated RT and anti-PD-1 combination (48). Since PD-1 is expressed by both PD-1^+^CD8^+^ T-cells and PD-1^+^ T-regulatory (Treg) cells, PD-1 blockade reactivates the effector effect of CD8^+^ T-cells and immunosuppressive Treg cells. Thus, it may be the ratio between the two, rather than the absolute levels of PD-1^+^CD8, which predicts response to PD-1 immune checkpoint inhibition (180).

While these data show a somewhat limited potential for certain immunotherapy regimens to induce a major pathologic response in HNSCC, the response was achieved shortly after administering it as monotherapy. Given the baseline differences in T-cells populations between patients and after treatment, it is possible that other regimens or combinations of immunotherapy will augment the immune response.

### 3.8. Neoadjuvant immunotherapy-SBRT combination

Although insufficient to signify a reversal of cancerous processes, the data reviewed so far points to possible positive trends in tilting the immune-cancer balance: Potential induction of immune response by SBRT, a possible benefit in combination therapies, a potential benefit, albeit slight, in starting immunotherapy in the neoadjuvant setting, and the supporting preclinical data. These logically lead to design studies that combine SBRT and immunotherapy in the neoadjuvant setting.

The combination of SBRT with durvalumab (anti-PD1) was assessed in the neoadjuvant setting of HPV-negative HNSCC in a phase I/Ib clinical trial (*n* = 21). The most common features were the oral cavity as the primary subsite (*n* = 18, 85.7%), T3 or T4 disease (*n* = 19, 90.5%), and node-positive disease (*n* = 14, 67%). The patients received one neoadjuvant dose of durvalumab, and the study was designed with an escalating radiation dose, starting 6 Gy ×2 to a maximum of 8 Gy ×3. For the 6 Gy ×3 or 8 Gy ×3 groups (*n* = 18), OS at 16 months was 80.1% (CI 95%: 62.0%–100%), and PFS and locoregional control were both 75% (CI 95%: 57%–99.8%). There was a positive association between 8 Gy ×3 dose and a better response (*p* = 0.07). In contrast, none of the recurred patients had a major pathologic or complete response (39).

Importantly, tissue samples obtained pretreatment (baseline) and at surgery (after neoadjuvant SBRT and durvalumab), demonstrated an increase in the CD103^+^CD39^+^CD8^+^ T-cells at baseline and at surgery among responders, similar to (80) and (181). Responders also had increased IFN-gamma within cytokine-producing T-cells and an increase in activated T-cells (PD1, CD69, Ki-67, and DNAM-1); responders had an increase in CD45RO^+^ memory T-cells, while non-responders had a less consistent pattern (39).

Gene expression analysis among responders revealed increased expression patterns associated with immune activation. In contrast, neither PD-L1 expression nor CPS scores correlated with response to durvalumab and SBRT (39). Although Treg cells in the TME were linked to decreased response to immunotherapy (182), a decrease in Treg cells was not a differentiator between responders and non-responders (39). Instead, the ratio between the amount of CD8^+^ T-cells to Tregs was correlated with response-to-treatment, with a decrease in total T-cells in non-responders, leading to a significant difference in the CD8^+^ T-cells to Treg ratio (39). The CD8^+^ T-cells to Treg ratio increased in patients receiving 8 Gy ×3 compared to lower doses of SBRT. Only by administering neoadjuvant 8 Gy ×3 were consistent MPR and CR observed in HPV-negative HNSCC concurrently treated with neoadjuvant durvalumab (39). This study is pivotal to the field as it systematically uncovers processes and trends underlying the resulting outcome. In addition to the optimal 8 Gy ×3 dose, it points to the CD8^+^ T-cells to Treg-cells ratio, baseline CD103^+^CD39^+^CD8^+^ T-cells, and T-cell activation markers as differentiators, and perhaps possible predictors of response.

In another phase Ib clinical trial, previously untreated locally-advanced HNSCC patients (*n* = 21) were treated with neoadjuvant SBRT (to GTV only) over one week. The doses studied were 8 Gy ×3 (24 Gy total dose) or 8 Gy ×5 (40 Gy total dose), with or without neoadjuvant nivolumab. Three cohorts (*n* = 16) were HPV positive, while the fourth was HPV negative (*n* = 5). All patients underwent standard-of-care surgery five weeks after SBRT, followed by adjuvant nivolumab for three months. The overall MPR was 86%, the CR was 67%, and 90% of patients were downstaged. The extent of resection was reduced in most patients, and no treatment-related surgical delays occurred. 20 of the 21 patients did not require adjuvant radiotherapy postoperatively. Delayed treatment-related adverse events were more common in the 40 Gy cohort. Of note, although major pathologic response was achieved in 86% of patients, partial radiologic response prior to surgery was evident in 10 patients, while 10 patients had stable radiographic disease. There was no correlation between the pathologic and radiographic response (64).

### 3.9. Summary—SBRT with immunotherapy

Currently, data on the combination of SBRT and immunotherapy in HNSCC is limited. So far, combining SBRT and immunotherapy in the metastatic setting has resulted in limited pathologic response. However, the published clinical trials indicate a more favorable outcome following neoadjuvant SBRT and immunotherapy. Of these two trials, the total dose of 24 Gy seems optimal, as a lower dosage led to a less favorable outcome (39), and a higher dose led to a similar outcome but a higher toxicity rate (64). The total RT dose, fractionation regimen, and the timeframe after completion of SBRT and before surgery may play a significant role in improving the pathologic outcome. Sparing the neck from the irradiation field may also play a significant role in allowing an immune response to develop after irradiating the primary site and administering immunotherapy.

Ongoing clinical trials of SBRT-immunotherapy combinations are summarized in Table 2.

**Table 2:** Selected combination SBRT-immunotherapy clinical trials in head and neck cancer.

NCT ID	Title	Phase	Patients enrolled	HNSCC population	Number of SBRT fractions	Total SBRT dose	Sequence of treatments
NCT04576091	Testing the Addition of an Anti-cancer Drug, BAY 1895344, With RT to the Usual Pembrolizumab Treatment for Recurrent HN Cancer	1	37	Recurrent unresectable	3		IT on day 1 of 1st cycle, BAY1895344 starting day 7 of 1st cycle, SBRT starting day 2–8 of 2nd cycle
NCT03283605	IT and SBRT for Metastatic HN Carcinomas	1/2	45	Metastatic			SBRT after 2 cycles of IT
NCT03212469	A Trial of Durvalumab and Tremelimumab in Combination With SBRT in Patients With Metastatic Cancer	1/2	54	Metastatic			IT starting day 1, SBRT staring day 15
NCT04830267	The Efficacy of Camrelizumab Plus SBRT in R/M HNSCC	2	70	R/M	3	27 Gy	IT starting day 1, SBRT starting day 14
NCT02684253	Screening Trial of Nivolumab With Image Guided SBRT Versus Nivolumab Alone in Patients With Metastatic HNSCC	2	65	Metastatic	3	27 Gy	IT starting day 1, SBRT starting day 14
NCT03546582	SBRT ± Pembrolizumab in Patients With Local-Regionally Recurrent or Second Primary HN carcinoma	2	102	Recurrent or new second primary			SBRT for 2 weeks, followed by IT
NCT04862455	NBTXR3, Radiation Therapy, and Pembrolizumab for the Treatment of Recurrent or Metastatic HNSCC	2	60	R/M			Injection of NBTXR3 on day 1, SBRT with concurrent IT starting day 3–8
NCT05136768	Sintilimab Combined With Chemotherapy and SBRT in Limited Metastatic HNSCC	2	50	Limited metastatic			IT and chemotherapy starting day 1, SBRT starting after at least 2 cycles of IT and chemotherapy
NCT03313804	Priming Immunotherapy in Advanced Disease With Radiation	2	57	Metastatic		30 Gy	IT starting day 1, SBRT starting day 1–14
NCT03635164	RT With Durvalumab Prior to Surgical Resection for HPV Negative Squamous Cell Carcinoma	1	21	HPV negative resectable	2 (escalate to 3)	12 Gy (escalate to 18 Gy)	Neoadjuvant IT + SBRT, followed by surgery at 3–6 weeks after SBRT
NCT05053737	RT in Combination With Atezolizumab Prior to Surgical Resection for HPV Unrelated HNSCC	1/2	46	HPV negative	3	24 Gy	Neoadjuvant IT + SBRT, followed by surgery
NCT04938609	Neoadjuvant Immunoradiotherapy in HN Cancer (NIRT 2-HNC)	2	28	Stage III-IVa HPV negative	3	24 Gy	Neoadjuvant IT + SBRT, followed by surgery at week 7
NCT03618134	SBRT and Durvalumab With or Without Tremelimumab Before Surgery in Treating Participants With Human Papillomavirus Positive Oropharyngeal Squamous Cell Caner	1/2	82	HPV positive resectable oropharyngeal			Neoadjuvant IT + SBRT, followed by TORS and neck dissection between weeks 6–8

HN, head and neck; HNSCC, head and neck squamous cell carcinoma; IT, immunotherapy; R/M, recurrent or metastatic; RT, radiation therapy; SBRT, stereotactic body radiation therapy; TORS, transoral robotic surgery.

## 4. New players in immunotherapy and radiotherapy

### 4.1. Cytokines

Checkpoint inhibitors such as anti-CTLA-4 and anti-PD-1 are clearly the major players in cancer immunotherapy, but they are the second phase of FDA-approved immunotherapies for cancer. The first phase was recombinant cytokine therapies; some are still in use. The anti-tumor activity of recombinant IL-2 led to FDA approval for patients with metastatic kidney cancer in 1992 and metastatic melanoma in 1998, and high-dose recombinant IL-2 is still in clinical practice (183). The toxicity of high-dose IL-2 and relatively low response rates limit its clinical use to specialized centers and some community hospital programs (183). However, it can generate clinically meaningful and durable responses and remains part of published treatment guidelines for both melanoma and renal cancer (184, 185). An initial phase I study demonstrated that SBRT combined with high-dose IL-2 resulted in an objective response of 71% in previously untreated patients with metastatic melanoma and 60% in renal cell carcinoma (66). There was no increase in the toxicities associated with high-dose IL-2 and no dose-limiting toxicities associated with radiation. A subsequent phase II randomized study showed an improved disease control rate in patients receiving SBRT combined with high-dose IL-2 compared to high-dose IL-2 alone (65). While the combination therapy was similar to the phase I results, an unexpectedly strong response in the IL-2 alone group limited the ability to detect an improvement with SBRT. This may have been influenced by dramatic improvements in treatment options for these patients in the past decade, including BRAF-targeted therapies and prior anti-PD-1 and anti-CTLA-4 treatments. Retrospective analyses suggest a higher-than-anticipated response to anti-PD-1 following IL-2 (186). Preclinical studies suggest novel IL-2 formulations can enhance immune activity with limited toxicity, and that these agents synergize with radiation therapy in preclinical models (187). The response rate could be increased through combination with innate adjuvants and correlated with antigen-presenting cells maturation (188), suggesting that improving antigen cross-presentation in combination with antigen release and T-cell support could provide multifaceted support of anti-tumor immunity.

Similarly, various cytokines within the Type I interferon (IFN) family have been studied as adjuvant therapy for cancer in the past 30 years. Treatment of patients with type I IFN resulted in improved recurrence-free survival (189, 190) but not OS (191). Preclinical studies in pancreatic cancer have shown improved outcomes with type I IFN and chemotherapy in pancreatic cancer (192, 193). However, clinical studies suggested that while patients receiving type I IFN and adjuvant chemoradiation showed improved outcomes (194), the use was limited by high-grade toxicity in 85%–90% of patients (195, 196). To control for systemic toxicity while sustaining tumor effects, type I IFN can be injected into the local tumor environment to generate tumor control (197) and can be engineered to accumulate in the vicinity of cancer cells using immune conjugates (198, 199). However, a range of alternative therapies has been developed to induce type I IFN in the tumor environment through local administration, improving the *in-vivo* efficacy and toxicity profile [reviewed in (20)]. Examples of these will be discussed below.

### 4.2. Innate adjuvants

The use of innate adjuvants to support the immune response to RT has been widely reviewed (20, 21, 113, 114). This is based in part on a wide range of studies using exogenous adjuvants injected into tumors to improve radiation-mediated control of tumors. For example, an important series of studies demonstrated that single fraction and fractionated RT regimens resulted in improved local control when combined with CpG in a mouse fibrosarcoma model (200, 201). These studies demonstrated that the 50% tumor cure dose for fractionated radiotherapy is reduced from 83.1 to 23 Gy when combined with CpG. As discussed above, more recent studies have identified that endogenous innate adjuvants that stimulate the STING pathway are generated by RT and are a critical component of the immune effects of radiation (56, 57, 202). Exogenous administration of STING ligands also synergizes with radiation to control tumors in preclinical models (203), and an array of methods to deliver STING ligands and similar innate therapies have been developed (204–206). However, these discoveries have proven difficult to translate, given the limited efficacy of STING ligands in clinical trials (207, 208). Considering the high potency of STING ligands in preclinical models and the lack of potency in patients, these data suggest either a problem in how these agents are translated to clinical use, or a fundamental limitation in the murine preclinical models used to develop these agents (50). Extensive further study is ongoing in this area.

### 4.3. Myeloid-targeted agents

As discussed above, while exogenous adjuvants can synergize with RT to control tumors, cancer cells killed by RT can provide endogenous adjuvants such as STING ligands (56), heat shock proteins (209–211), HMGB1 (212, 213), and calreticulin (214). However, macrophages in tumors are generally polarized such that they respond to TLR ligands with an M2 pattern of response by secreting cytokines such as VEGF, IL-10, and TGFβ (215–217). In addition, exposure of unpolarized macrophages to irradiated cancer cells can drive macrophages to become M2 polarized (97, 215, 218, 219), regardless of any adjuvants released. Since myeloid cells can be an obstacle to RT, there is a range of strategies focused on eliminating these cells or preventing M2 polarization [reviewed in (99)]. One such approach has been to target CSF1R, which drives macrophage differentiation and supports macrophages in peripheral tissues. CSF1 or CSF1R inhibition with blocking antibodies or small molecules has synergized with both chemotherapy and RT to control tumors (220, 221). Despite improved responses, this approach has a marginal effect and has not been shown to result in tumor cures.

As an alternative to macrophage depletion, targeting the pathways that drive M2 differentiation following macrophage interaction with dying cells has provided stronger impacts. Blocking phosphatidylserine (PS) (222), milk fat globulin E8 (MFGE8) (223), and Mertk (224, 225), have all altered macrophage differentiation following exposure to dying cells, and resulted in improved control of tumors. Mertk is a particularly relevant target since it is the signaling component downstream of PS ligation by Gas6 (226) and MFGE8 ligation by integrins (227), as well as complement-mediated opsonization of dying cells via C1q (228). Importantly, Mertk blockade combined with radiation can also be improved in resistant tumors by additional therapies such as TGFb inhibition or checkpoint regulators, which can permit control of distant tumors (99, 224, 225, 229), suggesting it is a good target to overcome macrophage suppression following radiation (99).

Recent studies have highlighted CD47 as a novel phagocytosis-related target in cancer therapy. CD47 binds SIRPa, where SIRPa is predominantly expressed on macrophages and some myeloid subpopulations, while CD47 is expressed on most cells, and particularly on hematopoietic cells and red blood cells (230). CD47 expression prevents phagocytosis of red blood cells (231). CD47 expression varies on immune cells through their activation, and high-level expression of CD47 on acute myeloid leukemia (AML) cells was associated with a worse prognosis (232). Antibodies blocking human CD47 on AML cells transplanted into immunodeficient mice resulted in limited engraftment of the human cells due to increased phagocytosis by host cells (232). In patients, where anti-CD47 can bind normal cells as well as cancer cells, most patients exhibit hematological toxicities with 100% receptor occupancy on red blood cells observed at doses above 1 mg/kg (233). Novel CD47 antibodies are in development that can potentially limit toxicity, but an alternative is to target the SIRPa molecule on myeloid cells. In immunocompetent preclinical models, the addition of anti-SIRPa to radiation resulted in improved control of tumors compared to either agent alone, and compared to anti-CD47 combined with RT (234). Further improvements in local and distant responses could be made by adding anti-PD1 (234), indicating again that myeloid targeting works well in combination with T-cell targeted therapies to improve radiation outcomes.

To provide a degree of certainty in selecting CD8^+^ T-cells to become long-lived memory cells, the immune system uses multi-factor authentication that depends on the presence of innate adjuvants as well as distinct CD4^+^ and CD8^+^ T-cell antigenic epitopes. These signals are integrated via dendritic cells, which traffic to lymph nodes in the presence of adjuvant and where CD4^+^ T-cells license dendritic cells via CD40-CD40l interactions to optimally activate CD8^+^ T-cells to cross-presented antigen (102–104). In the absence of CD4^+^ T-cells, effector CD8^+^ T-cell responses to infectious agents can still be generated, but memory responses are generally decreased (235, 236). CD40-CD40l signals are necessary to develop the T-cell immune environment of tumors, and this is, in turn, necessary for tumor control by radiation and immune checkpoint inhibitors (79). Therapeutically, CD8^+^ T-cell memory can be generated in the absence of CD4^+^ help by providing polyIC and anti-CD40 antibodies (237), thus, exogenously providing the DC adjuvant and the critical aspect of CD4^+^ help (102). Anti-CD40 treatment has been shown to improve responses to RT in a range of preclinical models (238–240). Anti-CD40 treatment has resulted in some on-target toxicity in patients (241), so novel approaches are in development to target this agonist. Locally administered anti-CD40, designed to slowly release into the tumor-draining lymph node, has shown an equivalent single agent response as systemic delivery and decreased toxicity (242), suggesting that targeted CD40 therapies have the potential to improve the use of this agent. A distinct sustained release system, providing both anti-CD40 and anti-PD1, has shown synergy with radiation in preclinical models (243), and antibody alone directly injected into tumors has shown synergy with radiation in preclinical models (244). Alternatively, a dual fibroblast and CD40 targeting antibody has been developed, which shows synergy with RT in preclinical models (245). These data are interesting since they suggest that DC help is relevant in the tumor environment rather than following trafficking to the lymph node, as has been shown for endogenous T-cell responses following radiation (30, 31). Importantly, as with other myeloid-targeted therapies, anti-CD40 therapy has been shown to be a strong partner for T-cell targeted immunotherapies (246), suggesting that these treatments can be layered to optimally treat tumors that have limited pre-existing immunity.

### 4.4. Metabolic targets in the tumor environment

The unique environment of a growing tumor can engender a range of unusual metabolic conditions that are a target for therapy. In general, the active proliferation of cancer cells can lead to the depletion of metabolites along with hypoxic conditions of high growth outstripping vascular supply (247). Many of these metabolic conditions are immunoregulatory, and critical pathways can impair immune responses in the tumor environment. For example, prostaglandin E2 (PGE2) is a long-defined feature of the tumor environment that suppresses the immunostimulatory activity of DAMPs on DC and macrophages (248–250). Radiation increases PGE2 production by irradiated tumor cells and tumor stroma cells, and this impacts cancer cell repopulation and results in poor therapeutic outcomes (251).

Even positive features of cancer treatment can have negative metabolic consequences that impact outcomes. For example, signaling through type I IFN signaling following treatment with radiation and exogenous adjuvants increases the expression of indoleamine 2,3-dioxygenase 1 (IDO1) (252), which can negatively regulate immune activation. IDO1 expression in tumors can promote tumor growth (253, 254), and patients with higher IDO1 expression have been shown to have worse outcomes (255). IDO1 impacts a range of immune cells in the tumor immune environment, including CD8^+^ T-cells and myeloid cells [reviewed in (254)]. Similarly, arginase induction in myeloid cells following RT of tumors can result in metabolic suppression of T-cells and limited T-cell control of irradiated tumors (256), and macrophages infiltrating tumors following immunotherapy can suppress T-cell control of tumors via arginase expression (100). These data suggest that targeting this specific metabolic feature can enhance the radiation control of tumors.

Recently, several studies have pointed to purinergic signaling as an important target in cancer [reviewed in (257)] and following RT. Extracellular ATP concentrations are regulated by the ectonucleotidases CD39 and CD73, which are themselves regulated on immune cells in tumors (80, 258, 259). ATP is hydrolyzed to ADP and AMP by CD39, and AMP is further hydrolyzed to adenosine by CD73. Adenosine can, in turn, generate anti-inflammatory and immunosuppressive effects in the tumor environment, including promoting a tolerogenic phenotype in DC (260), and directly inducing T-cell anergy and Treg differentiation (260–262). While CD39 is enriched on tumor-specific T-cells in tumors (80, 263) as well as Treg cells, increased expression of CD73 is associated with a poor prognosis in a range of tumors (264–266). Importantly, hypoxia and inflammation in the tumor can upregulate the expression of CD39 and CD73, resulting in radio-resistance (267, 268). Thus, blocking CD73 can improve the response to radiation in preclinical models (269). Similarly, a range of studies have demonstrated that targeting adenosine metabolism and purinergic signaling has improved immune control of tumors (261, 270), the response to radiation in preclinical models (271, 272), and a range of related approaches are in clinical development (273). Again, given that these factors limit T-cell control of tumors, these can be layered with T-cell-targeted immunotherapy to improve tumor control.

## 5. The effect of heterogeneity on response to radiotherapy

### 5.1. Signaling mechanisms and intratumor heterogeneity

**Figure 2:**
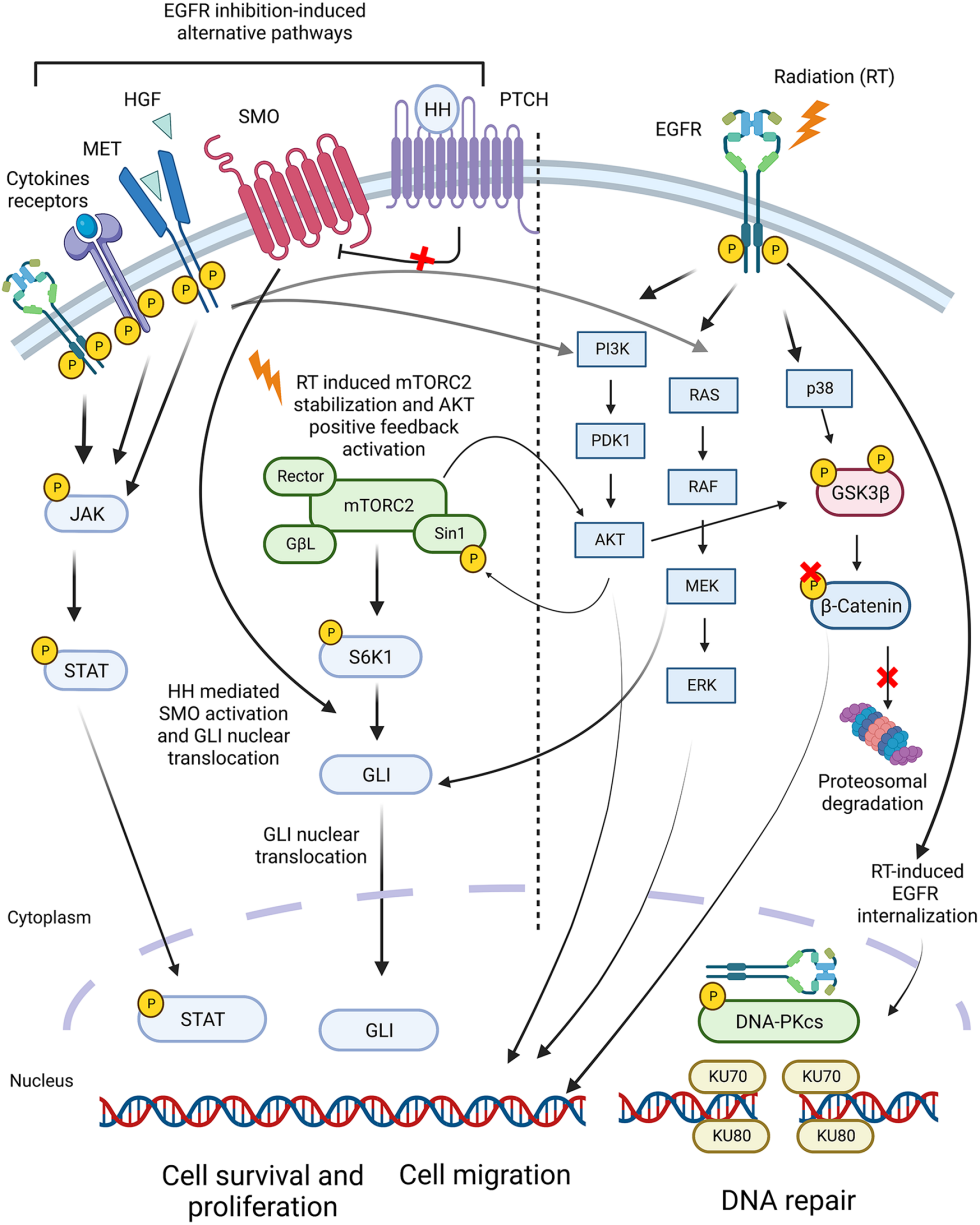
Signaling pathways in HNSCC involved in the development of resistance to radiotherapy. The Epidermal Growth Factor Receptor (EGFR) was found as one of the central components of resistance to RT due to activation of pro-survival mechanisms, such as pAkt/MAPK or DNA repair pathways. However, combined inhibition of EGFR with RT did not improve HNSCC response to RT. This is due to alternative pathways activated in response to EGFR inhibition, such as HGF/MET, JAK-STAT, and Hedgehog pathways. Inhibition of these pathways, along with EGFR, improved the response of HNSCC to RT in preclinical studies.

Recently, advances in molecular biology and a better understanding of the molecular mechanisms underlying HNSCC have resulted in the development of targeted therapy to boost radio-sensitization. A few agents are being studied, including anti-epidermal growth factor receptor (EGFR). EGFR is overexpressed in over 90% of head and neck tumors and is linked to poor prognosis and increased tumor growth and metastasis. Moreover, EGFR was found as one of the critical components of resistance to RT (274, 275) through activation of downstream pro-survival mechanisms, such as pAkt/ MAPK or DNA repair pathways, when it internalizes to nuclei and activates DNA-PK (274) (Figure 2, right panel).

However, the combined therapy of anti-EGFR with RT did not significantly improve treatment response due to alternative pathways activated in response to EGFR inhibition, such as HGF/MET, JAK-STAT, and Hedgehog pathways. Indeed, inhibition of these pathways along with EGFR improved HNSCC response to RT in preclinical studies (274, 276, 277) (Figure 2, left panel).

Recent data suggest that the activation of alternative pathways is often patient-specific (278). For example, certain HNSCC malignancies can activate cMet pathways along with EGFR^+^ processes, whereas others may harbor EGFR^+^ and Src^+^ distinct subnetworks. Hence, in this example, two different drug combinations should be selected to treat these HNSCC malignancies. Analyzing proteomic and phospho-proteomic alterations, 61 distinct tumor subtypes were found in a cohort of 203 HNSCC patients (278), suggesting a high level of intertumor heterogeneity, and thus an urgent need for personalized therapies.

Complex, often spatial-dependent (279–281) interactions between cancer cells, the immune system, microbiome, and additional individualized elements in the TME, contribute to the intratumor cellular diversity, thereby complicating the intra- and intertumor heterogeneity of HNSCC. Intratumor processes and communication with the TME generate constant selective pressure, which promotes continuous diversification of malignant and nonmalignant compartments of TME, thereby increasing a degree of intratumoral heterogeneity, aggressive disease progression, and resistance to treatments (282). An example of spatial heterogeneity in HNSCC was provided by Forum et al., who demonstrated that the invasive leading edge of primary tumors was occupied by cancer cells expressing epithelial-mesenchymal transition (EMT) signature genes, while cancer cells located within the core of the tumor did not show EMT transcription factors (283). In addition to being a factor involved in intratumor heterogeneity, EMT is also associated with, and is widely considered, a potential cause of drug resistance, invasion, and metastasis (284).

Non-homogeneous distribution of immune cells within solid tumors is another example of non-homogeneous intratumor evolution, which may contribute to immunotherapy-RT resistance. For example, it was shown that hypoxia might drive the localization of tumor-associated macrophages. M1 macrophages, a subtype displaying an anti-tumor phenotype, were found mainly in normoxic areas approximate to blood vessels, while M2 macrophages, the protumor subtype, were more dominant in hypoxic areas in lung cancer (285). The intratumor diversity of TME, including hypoxia and cancer-associated fibroblasts (CAF), was found to have an essential role in developing the M2 macrophage subpopulation in head and neck cancers (286) and development of the HNSCC resistance to RT and immunotherapy.

Thus, an in-depth understanding of intratumoral heterogeneity, along with the changes occurring in response to RT/immunotherapy, can be crucial to the design of individualized therapy for head and neck cancer patients.

Studies that capture and target intratumor evolution are underway in other cancer fields. For example, Alkhatib et al. have shown that RT resistance may occur due to the evolution of the intratumor cellular populations in response to RT, towards a more radio-resistant molecular composition. The study characterized evolving processes in triple-negative breast tumors in response to RT and found two different HER2 and cMET-positive cellular subpopulations, which have been expanded in the resistant tumors. Simultaneous inhibition of HER2 and cMET receptors sensitized the tumor responses to RT (287). This research suggests that a similar approach, providing an accurate molecular characterization of tumors undergoing RT, may be beneficial for HNSCC as well.

### 5.2. Computational tools to resolve heterogeneous cancer responses

Growing evidence for the extensive intertumor and intratumor heterogeneities in HNSCC, and their potential influence on different types of treatments, led to the development of quantitative approaches addressing the challenge of accurately classifying cancer patients (or cells within a tumor) into distinct subgroups.

For example, in a phase I trial of neoadjuvant SBRT and anti-PD1 prior to surgery, responders exhibited higher levels of proinflammatory cytokines IFN gamma and TNF alpha, and an increase in circulating memory T-cells, while non-responders had more prominent TGF beta, IL-17A, and DNAM-1 expressing myeloid populations (39). While each attribute may constitute a potential biomarker for patient response, accumulating knowledge in precision oncology suggests that quantitative strategies should classify cancer patients based on specific features of their altered protein-protein networks rather than on overexpression or mutation of a specific biomarker. Once differences and similarities in these networks are identified and found to differentiate between individuals, protein hubs from each subgroup-specific network can be transformed into practical clinical solutions. Namely, an accurate stratification will significantly increase response to existing therapies or lead to the development of new therapeutic strategies.

Machine deep-learning algorithms play an essential role in these efforts. These techniques attempt to derive a general rule from the input proteomics (or genomics) data, and then to “allocate” each patient into a specific subgroup according to this rule. This classification can generate a prediction of drug sensitivity for a given patient.

Examples of machine-learning methods include clustering algorithms, such as the K-nearest neighbor algorithm, K-Means algorithm, and support vector machine (SVM). These calculate from the experimental (such as protein expression data) how similar the samples are in terms of protein expression alterations, and then calculate the distance between every two samples. These machine-learning algorithms, and others that will be described next, were frequently used for early diagnosis and prevention of head and neck cancer (288). Recently, they have been applied to differentiate responders from non-responders to anticancer drugs in oral (289) and additional cancer types (290), as well as predictors for cancer subtypes (291) and survival of cancer patients (289, 292).

Additional examples include Bayesian Networks classifiers, which use conditional probabilities to calculate the probability of sample B to belong to a particular cluster, given a set of samples features, e.g., a particular protein expression signature (293), or Decision Tree algorithms, which sequentially use data variables to compute the sequence of branch choices. These types of machine-learning algorithms were used to predict clinical outcomes (289, 294) through the identification of essential gene lists or clinical features associated with survival (Bayesian Networks classifiers) (295, 296), or to predict aggressive behaviors of tumors via DNA methylation differences (Decision Tree algorithms) (289, 297).

Neural network-based models are an example of the most popular deep learning algorithms employed today to study cancer systems. Similar to the connections of neurons and synapses found in the brain, the algorithm processes several “layers” of data, each one using increasingly complex analysis than the previous one. In oral cancer, this approach was applied, for example, to transcriptomic data to identify different immune subtypes (298). Additional discussion on machine-learning and deep-learning approaches can be found in (293, 299).

### 5.3. Physics-based models

While predictions based on probabilities identify abundant patterns in a patient population, and then assume that a patient possessing this pattern should respond to a drug in a certain way, physical approaches base their predictions on the stabilities of systems. They are derived, for example, from free energy quantifications for each state of the system (300–304). According to the basic physicochemical laws, spontaneous transitions from a higher to lower free energy state occurs in a system, and thus would define a direction of spontaneous change in the course of any process, including pathological process. Therefore, identifying stable and unstable states in tissues, including cancer tissues, should allow us to predict different phenotypes and recommend how those phenotypes can be manipulated.

This notion inspired us and others to implement thermodynamic-based approaches in cancer (302, 305, 306) and extend it further to the field of personalized medicine (301, 307). A series of works demonstrated that the thermodynamic-based strategies could provide detailed information not only on the central oncomarkers (oncological biomarkers) or common patterns characterizing a particular subgroup of patients, but also on the role of each oncomarker in the patient-specific ongoing processes (301, 307, 308). Once a patient-specific set of unbalanced processes is resolved, then a prediction on how a tumor-specific imbalance should be targeted is readily provided (287, 307).

Using unbalanced processes resolved in each HNSCC malignancy, we have recently demonstrated how they can be used to design patient-specific drug combinations. Jubran et al. have shown that combining the anti-EGFR inhibitor with additional, patient-specific targeted drugs, results in higher efficacy than the anti-EGFR monotherapy. Using *in-vivo* studies, Jubran et al. demonstrated that the resistance to anti-EGFR therapies could be inhibited when the unbalanced processes, occurring in addition to the EGFR^+^ processes, are resolved and targeted simultaneously in a patient-specific manner (278). Moreover, the study provided evidence that patient-specific drug therapies can also increase the potential of T-cell activation. These results suggest that resolving the individualized unbalanced processes, basal or induced to a specific type of therapy, provides an essential step towards accurately designing patient-specific drug combinations (278, 287, 307, 309).

In summary, quantitative, statistical, or physicochemical approaches provide great promise for accurate patient stratification to individualize diagnostics and treatments. Future methods will likely focus on integrating omics, clinical data, and image data, using multimodal learning (310, 311) to reveal novel molecular aberrations that differentiate certain groups of tumors from others. One of the central clinical challenges would be to find and implement a strategy that addresses each tumor individually, and provides a complete molecular characterization to any new patient. In particular, to those who do not necessarily possess the features learned from the previous patient populations.

## 6. Conclusions

The introduction of immunotherapy, targeted therapy, and hypofractionated RT into the field of head and neck cancer has opened new possibilities for treatment. Recent developments in diagnostic and computational methods allow better characterization of the patients and tumors and assess treatment outcomes. However, adapting the appropriate regimens to patients, and predicting their outcomes have not matured into practical tools.

RT, once regarded only as a means to kill cancer cells directly, is transforming into a multilayered tool to impact the immune response. Preclinical studies and clinical trials have uncovered the impact of dose adjustment, hypofractionation, and lymph node sparing to allow and even augment systemic immunity against cancer cells. A better understanding of immune cells trafficking, response-to-treatment, and implementation of *in-silico* methods, will facilitate treatment design and prediction of response.

FDA-approved immunotherapy and targeted therapy in head and neck cancer are still limited to a narrow set of agents, with most published clinical trials resulting in less-than-optimal results. However, the natural propagation of clinical trials from the recurrent and metastatic setting into the upfront and even neoadjuvant settings have resulted in encouraging data upon which ongoing and future clinical trials will follow. Current data show that RT fractionation and the sequence of surgery, RT, chemotherapy, immunotherapy, and targeted therapy should be designed to allow systemic immunity to develop. In particular, while upfront surgery followed by an immunotherapy-RT combination led to worse local control and immune response, neoadjuvant immunotherapy-RT combination followed by surgery resulted in better local control and systemic immunity. The optimal RT dose and its combination with immunotherapy and targeted therapy are still under investigation.

In conclusion, in recent years, there have been major advancements in the field of head and neck cancer. Although mostly confined to clinical trial settings, the evolution of an immune-directed approach to treat head and neck cancer seems well underway. Basic research studies and clinical trials are key to advancing toward the next milestone. And then to the next.
